# The resilience of distally based sural fascio cutaneous flap in soft-tissue defects of lower limb and ankle: a prospective study

**DOI:** 10.1186/s13018-025-05575-9

**Published:** 2025-02-27

**Authors:** Mohamed Romeih, Ahmad Abdulwahab Al-Shamy

**Affiliations:** 1 Orthopedic Surgery, Orthopedic Surgery Department, Faculty of Medicine, Tanta University, Tanta, Al-Gharbia Egypt; 2 Orthopedic Surgery, Faculty of Medicine, Tanta University, Tanta, Egypt

**Keywords:** Lower extremities, Reverse sural flap, Soft tissue defects

## Abstract

**Background:**

Managing large soft-tissue defects in the distal lower extremities remains challenging for orthopedic surgeons, particularly in elderly patients with comorbidities. This study evaluates the clinical outcomes of the reverse sural flap (RSF) for reconstructing soft-tissue defects in the distal leg, heel, foot, and ankle.

**Methods:**

This prospective study was performed on 52 cases aged from 18 to 60 years old, with either post-traumatic or post-surgical soft tissue defects situated between the distal third leg and the mid-metatarsals of the foot and underwent RSF surgery.

**Results:**

The mean hospital stay was 8.4 (± 3.24) days. All patients experienced the healing of their soft tissue coverage. During the follow-up, ten patients had complications: Ankle stiffness occurred in 2 (3.85%) patients, marginal necrosis in 2 (3.85%) patients, superficial infection occurred in 2 (3.85%) and delayed healing in 4 (7.69%) patients.

**Conclusion:**

The RSF is a reliable and practical option for reconstructing significant and distal soft tissue defects in the lower extremities, with acceptable complication rates and surgical durations. Complications were found in 10 cases in the form of ankle stiffness occurring in 2 (3.85%) patients; marginal necrosis occurred in 2 (3.85%), superficial infection occurred in 2 (3.85%) patients managed with dressing, debridement, delayed healing occurred in 4 (7.69%) patients and. The hospital stay’s mean value (± SD) was 8.4 (± 3.24) days.

## Introduction

In orthoplastic surgery, addressing soft tissue defects in the lower extremities can pose significant challenges, particularly in traumas, chronic wounds, or post-surgical complications. The reverse sural flap (RSF) has emerged as a reliable and versatile option for reconstructing these defects, offering several advantages over other techniques [1].

Flap surgery is a surgical method employed in plastic and reconstructive procedures, where tissue from a designated donor site is transferred to a recipient site, maintaining a secure blood supply throughout the process [2]. In cases where the remaining tissue is insufficient to support a graft, an RSF can be performed to cover defects [3].

The RSF is based on the reverse flow of the sural artery, which supplies the skin and soft tissue of the lower leg and foot [4, 5].

The RSF offers a relatively large surface area for coverage, making it suitable for addressing moderate to significant soft tissue defects [1]. The flap can be raised as a fasciocutaneous or fascioadipofascial flap, depending on the requirement for bulk and contour. This versatility allows for tailored reconstruction to address a wide range of defects, including those involving exposed bone, tendons, or hardware [6].

The RSF has the advantage of minimizing donor site morbidity. The donor site can typically be closed primarily or covered with a split-thickness skin graft, resulting in a well-concealed scar and minimal functional impairment [7].

The RSF’s simplicity makes it accessible for surgeons with varying levels of experience [8, 9], the donor site morbidity is minimal, and it is performed in a single stage, which can help minimize the overall duration of the surgical procedure and reduce the hospital stay for patients [10]Thus, this work was conducted to evaluate the clinical outcome and usefulness of the RSF for covering soft tissue defects of the distal third leg, heel, foot, and ankle.

## Patients and methods

This prospective study was conducted on 52 patients aged 18 to 60, both sexes, with soft-tissue defects located between the distal third calf and the mid-metatarsals of the foot who underwent RSF surgery.

The study was conducted from December 2016 to December 2023 after approval from the Ethical Committee Tanta University Hospitals (approval code: 36264PR458/12/23). The patient provided informed written consent.

Compromised peroneal artery or apparent lower extremity ischemia were excluded from the study.

All patients underwent complete history taking, clinical examination, laboratory investigations, and radiological investigations [arterial duplex US to confirm that the sural artery is intact, and Plain X-ray].

Before the surgery, a routine handheld Doppler US examination was conducted in every case to confirm the patency of distal perforators. In contrast, an anteroposterior (AP) and lateral plain X-ray were undertaken to exclude the possibility of concomitant fractures.

### Reverse sural flap

**Fig. 1:**
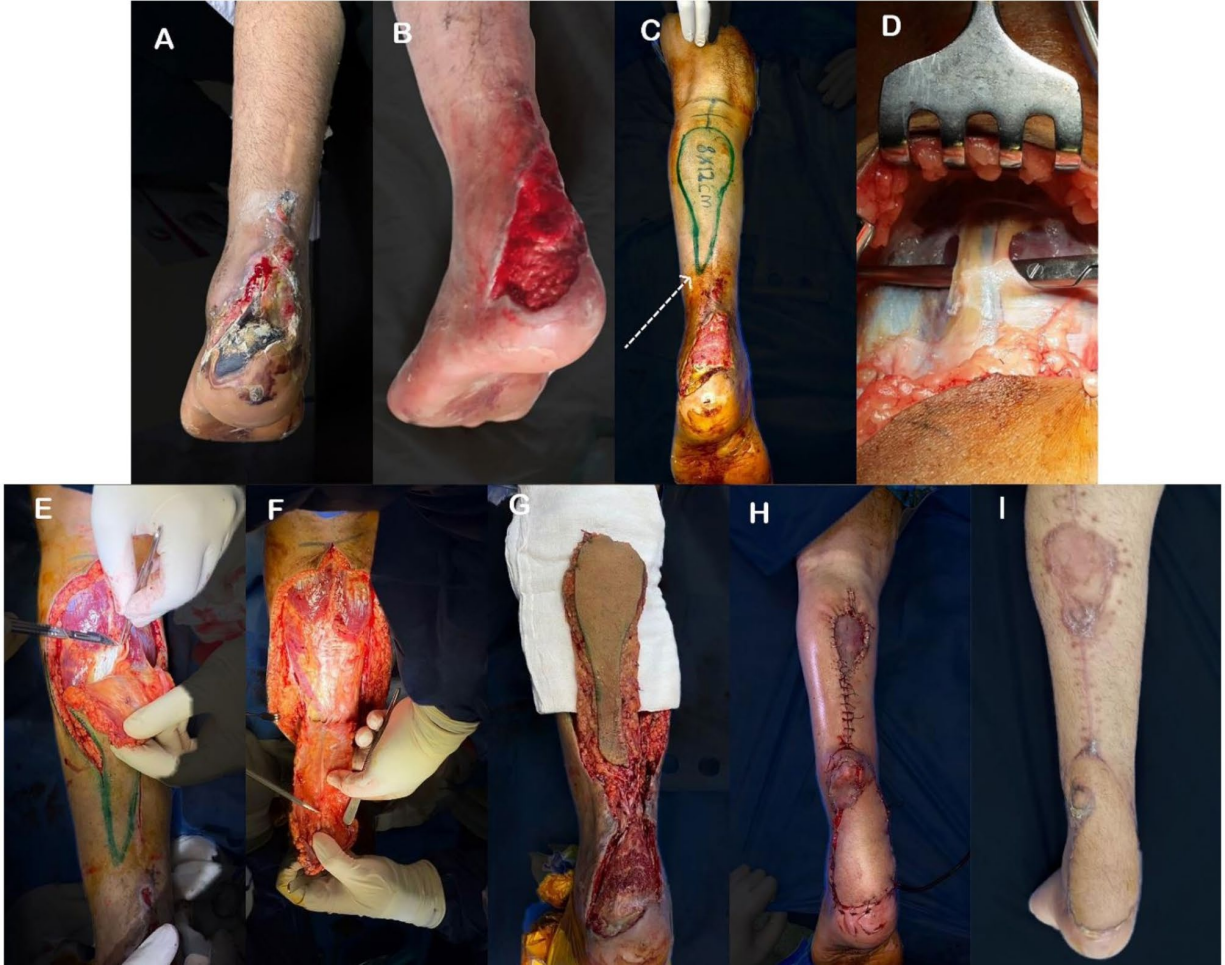
**A**) post-surgical wound dehiscence at heel with exposure of tendon Achilles, **B**) intraoperative picture after debridement, **C**) Planning and measuring the defect and corresponding flap with tail at the pedicle” dotted arrow,” **D**-**G**) harvesting the adipofasciocutaneous flap, **H**) after coverage of the defect and split-thickness skin graft on donor site, **I**) at 3 months follow up showing both donor and recipient sites

During a single-stage operation, and after ensuring that any existing infection was fully resolved, the patient was positioned in a prone or semi-prone position under spinal anesthesia, and the entire lower extremity was kept within the operative field. The thigh was prepared for skin grafting at the end of the procedure. A sterile tourniquet was utilized for better visualization. The initial step was to surgically debride the wound, measuring the defect and mapping out the flap. Figure 1A, B.

The flap entry point in the wound bed was determined to be typically situated where the flap can be rotated medially to fit the wound dimensions optimally. Figure 1C.

After determining the entry point, the lateral malleolus tip was detected and marked. A transverse line 5 cm proximal to the tip was drawn on the skin, serving as a reference point beyond which further distal dissection should not proceed. This mark represented the most distal boundary of the peroneal perforators and the pivot point of the pedicle and flap.

Then, the pedicle length was determined (by measuring the distance from the entry point at the wound bed to the midline of the leg and adding 1 cm to that measurement.). The ideal pedicle length allowed the flap to move into the wound bed without straining its blood supply and without being too long to avoid redundancy. For a 180-degree rotation, an extra 1.5 cm was added to ensure adequate length for a tension-free insect.

A pedicle width of 4 cm was preferred to minimize the risk of damaging blood vessels during the procedure, leaving 2 cm on each side of the midline. The dissection started by cutting the skin on the flap’s upper edge down to the deep fascia. The dissection began by incising the skin on the upper edge of the flap down to the deep fascia. The small saphenous vein and nerve bundles were situated in the center of the flap, close to the top. The artery and vein were clipped or ligated, and the sural nerve was identified and ligated. Figure 1D.

When dissecting the flap distally, care was taken to avoid severing the flap from the pedicle, which was located at the inferior margin of the flap. The flap was carefully elevated to maintain a clear, unbroken pedicle and dissected to the pivot point. The surgeon ensured the skin, subcutaneous tissue, and fascia were incised during dissection without beveling the margins. The paratenon over the gastrocnemius was left intact. Figure 1E.

The next step was to carefully separate the pedicle from the surrounding tissue along its length. Figure 1G Afterwards, the flap was turned to cover the defects and stitched into place, typically over-exposed deeper structures like tendons, bones, or joints.

An interpolated flap technique was employed, leaving the pedicle with a blood supply on the skin surface and a fasciocutaneous tail to protect and prevent kinking or tunneling beneath the skin or defect. If tunneling was performed, it was ensured that there were no constriction areas in the pedicle.

After positioning the flap, it was stitched into place and spaced 1–1.5 cm apart. Small donor sites were closed directly, while larger ones needed a skin graft. A skin graft was also applied to the flap’s base regardless of donor site size. Primary closure was performed when the donor site was small enough, such as with a small-sized flap. A split-thickness skin graft (STSG) was used for larger flaps to cover the donor site. In either case, STSG was necessary to cover the exposed pedicle. Figure 1H.

The flap area was covered with non-adhesive gauze and absorbent padding, while the regions surrounding the pedicle and flap were supported with bulky dressings. To reduce the risk of equinus contracture, a splint was positioned at the back of the limb.

Postoperatively, the patient was advised to bed rest with the leg raised for two days, during which time the flap was assessed for successful edge attachment and any signs of necrosis. If the flap showed signs of proper integration, the patient was fitted with a flexible dressing and a detachable splint positioned at the back of the leg before being sent home with instructions to maintain leg elevation for seven to ten days. Avoiding weight-bearing on the affected leg helps prevent venous congestion, which occurs when there is pressure on the leg that leads to poor blood flow and the pooling of blood, reduces pain and edema or swelling in the operated limb, providing greater comfort during the recovery process and allow the surgical site to heal correctly without disruption and minimizes the risk of complications.

Low-molecular-weight heparin (LMWH) was initiated 12 h after surgery. The typical prophylactic dose was 40 mg administered subcutaneously (SC) once daily, and in high-risk patients, the dose was adjusted to 1 mg/kg once daily. The treatment course typically lasts 6 to 11 days until the risk of deep vein thrombosis significantly decreases.

In cases where the flap was progressing well without signs of vascular issues or infection, the patient began Achilles tendon stretching and limb mobilization exercises.

We followed up with all the patients for 1 year. Complications such as ankle stiffness, distal necrosis, partial flap loss, superficial infection, and delayed healing were noted.

### Statistical analysis

The statistical analysis was analyzed using SPSS v26 software (IBM Inc., Chicago, IL, USA). The quantitative variables were displayed as the mean and standard deviation (SD), while the qualitative ones were displayed as frequency and percentage (%).

## Results

**Fig. 2:**
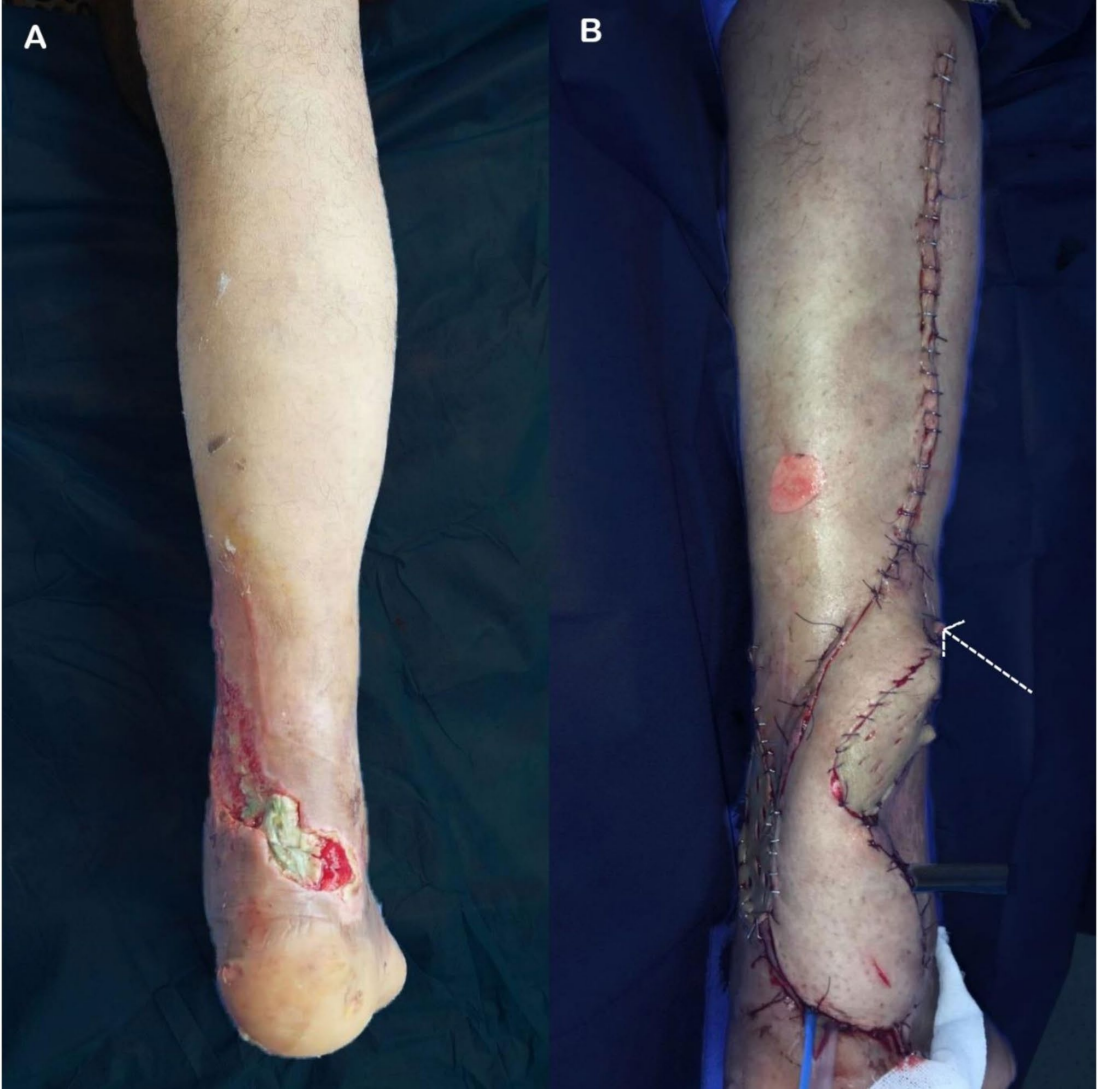
(**A**) post-traumatic exposure of avulsed tendon Achilles, and (**B**) after coverage with reverse sural flap noting the tail “dotted arrow” to protect the pedicle

**Fig. 3:**
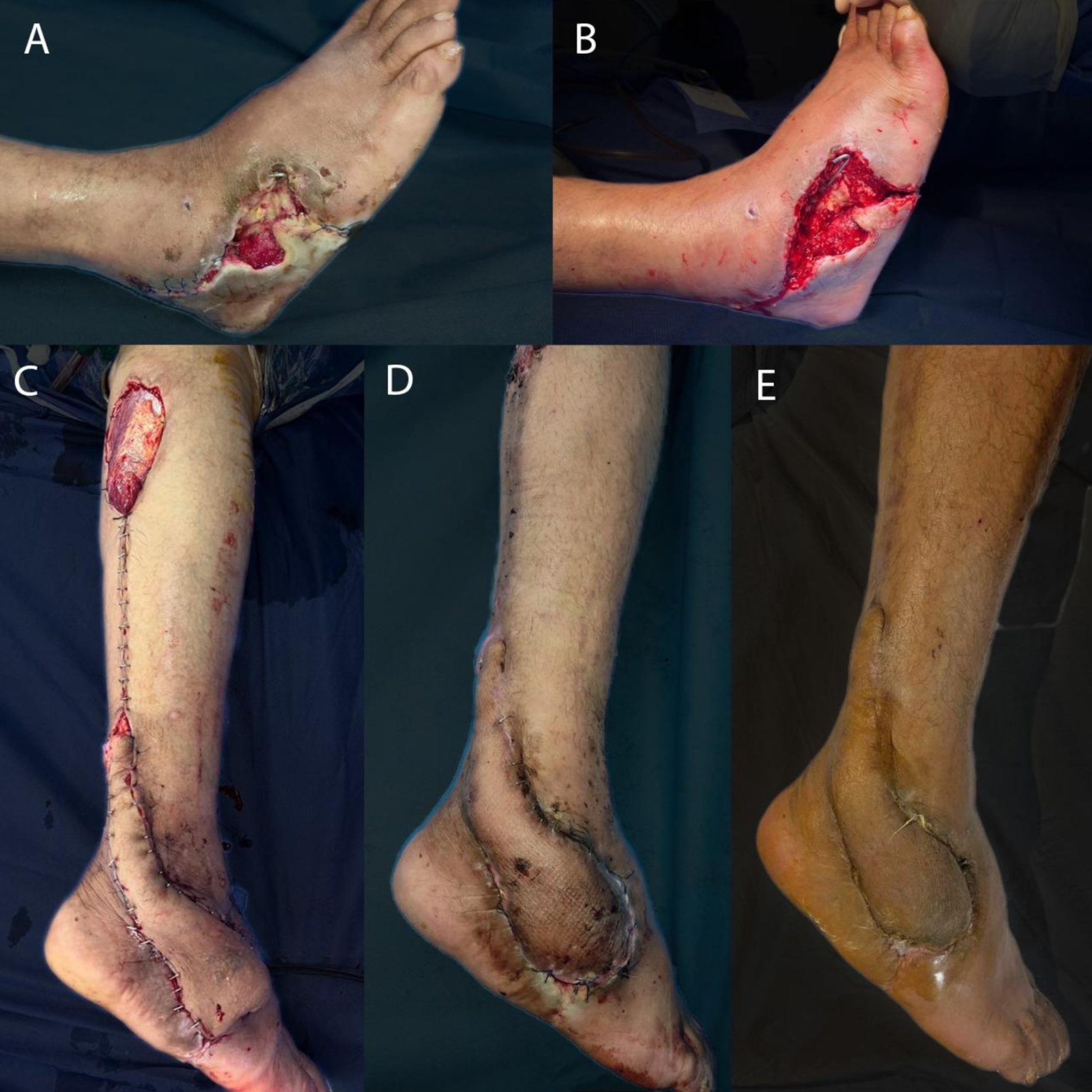
Intra-operative pictures of a case of infected wound dehiscence at the dorsolateral side of the foot after excision of giant cell tumor of the tendon sheath, **A**, **B**) before and after debridement, **C**) covering the defect with reverse sural flap, and **D**) at 1 month follow up, E) at 3 months follow up

**Fig. 4:**
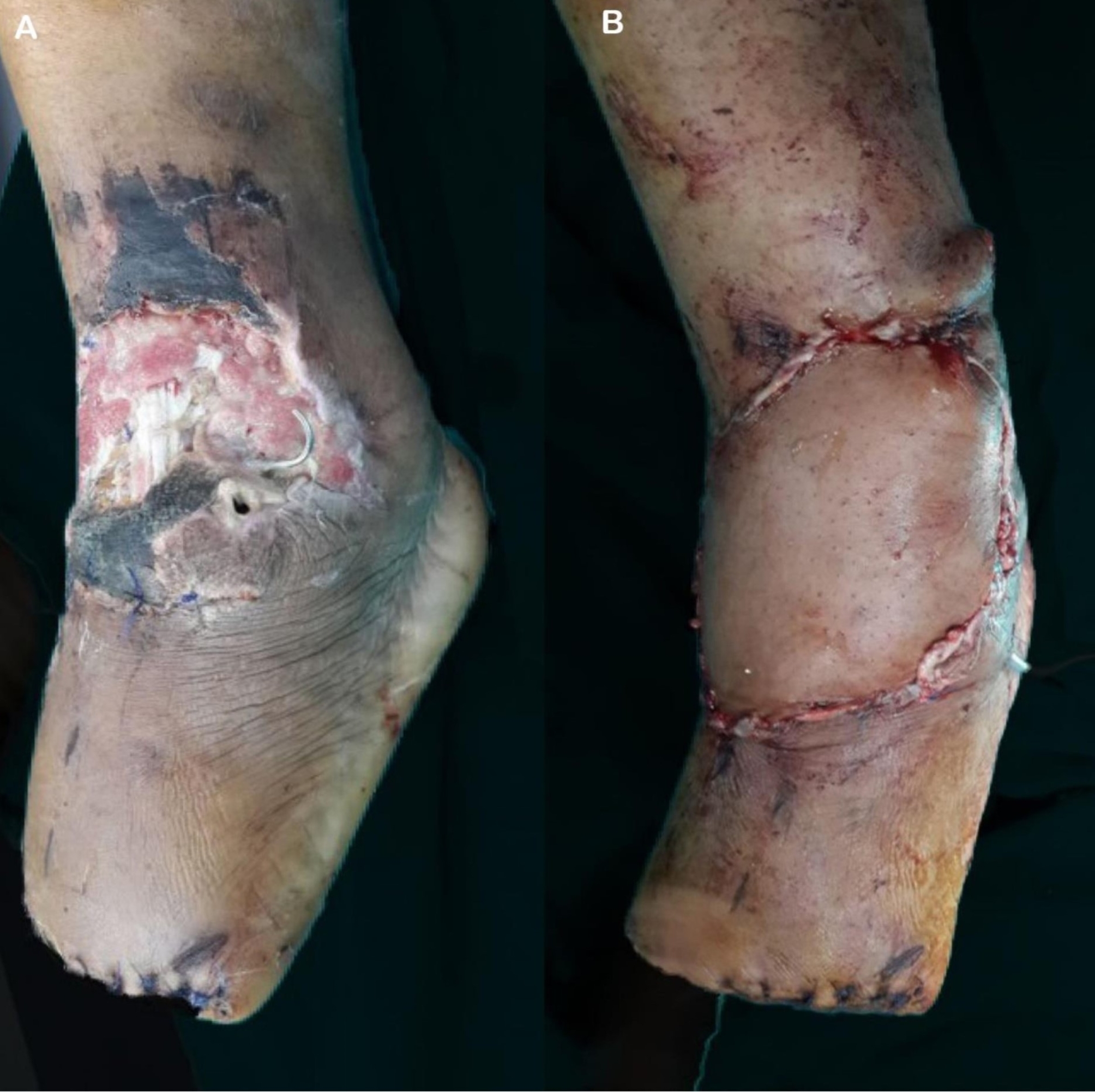
**A**): post-surgical wound dehiscence at ankle and dorsum of foot, and **B**): after coverage of the defect using reverse sural flap

The mean age was 48.6 years. Of the patients, 46 (88.46%) were males. 23 (44.23%) patients were smokers. Thirty-one patients had a comorbidity, more commonly diabetes mellitus, HTN, cardiac, and less commonly renal, hepatic patients. The mean flap size was 11.3 * 6.5 cm. The mechanism of injury was either post-traumatic (Fig. 2) or a more common post-surgical wound complication. Figures 3 and 4.

The site was ankle and foot in 21 (69.23%) patients, distal 1/3 leg in 22 (26.92%) patients, and heel in 9(1.92%) patients. The associated injuries were distal tibia fracture in 8 (15.38%) patients, malleolar ankle fracture in 4 (21.15%) patients, and skin loss after repair of Achilles tendon tear in 5 (9.62%) patients. The mean value (± SD) of the duration of surgery was 148.9 (± 41.55) min. Table 1.

**Table 1:** Demographic data and duration of surgery of the studied patients

		*N* = 52
Age (years)		39.1 ± 8.87
Sex	Male	46 (88.46%)
	Female	6 (11.54%)
Smoking		23 (44.23%)
Flap size (cm)		9.2 ± 5.1 * 6.5 ± 3.8
Site	Distal 1/3 leg	22 (69.23%)
	Ankle and foot	21 (26.92%)
	Heel	9(1.92%)
Associated injury	Distal Tibial fracture	8 (15.38%)
	Malleolar Ankle fracture	4 (21.15%)
	Skin loss after repair of Achelous tendon tear	5 (9.62%)
Duration of surgery (min)		115.6 ± 25.3

Data are presented as mean ± SD or frequency (%)

Complications were found in 10 cases in the form of ankle stiffness occurring in 2 (3.85%) patients; marginal necrosis occurred in 2 (3.85%), superficial infection occurred in 2 (3.85%) patients managed with dressing, debridement, delayed healing occurred in 4 (7.69%) patients and. The hospital stay’s mean value (± SD) was 8.4 (± 3.24) days. Table 2.

**Table 2:** Complications and hospital stay of the studied patients

		*N* = 52
Complications	Ankle stiffness	2 (3.85%)
	Marginal necrosis	2 (3.85%)
	Superficial infection	2 (3.85%)
	Delayed healing	4 (7.69%)
Hospital stays (days)		8.4 ± 3.24

Data are presented as mean ± SD or frequency (%)

## Discussion

The lower extremity has long been recognized as a region with compromised wound healing capabilities, and soft tissue defect reconstruction in this area is challenging and requires careful consideration and expertise [11]. The key principles of lower limb reconstruction involve matching the characteristics of the defect with those of the reconstructive tissue while also diminishing donor site morbidity, preserving main vascular structures, especially in elderly patients with comorbidities that put risk on the vascularity of the limb, and reducing operative and hospitalization time [12]. Perforator-based flaps are particularly a workhorse for this purpose [13]. RSF is a valuable local flap for reconstructing defects on the foot and ankle caused by trauma, chronic wounds, osteomyelitis, or burn scar contracture. Refining the surgical technique and patient selection can improve the success of RSF in reconstructing traumatic foot and ankle defects [14]. In our setting, where resources are limited, RSF has emerged as a reliable and safe option for soft tissue coverage.

In this study, the mean flap size was 11.3 * 6.5 cm. The site was the ankle and foot in 69.23%, distal quarter leg in 26.92%, and heel in 1.92%. The mean hospital stay was 8.4 ± 3.24 days.

Singh et al. [15] illustrated that the soft tissue defect site was distal 1/3rd leg in 73.8%, ankle medial aspect in 25%, lateral aspect in 6.25%, and heel in 6.25%.

Clivatti et al. [16] demonstrated that the injury was the ankle in 66.67%, the foot in 22.22%, and the calcaneus in 11.11%. The average hospital stay was 30.1 days. The more prolonged hospital stay reported in their study may be associated with the cause of injury as most patients (55.56%) had wounds resulting from motor vehicle accidents. In comparison, 33.33% had developed chronic wounds following traumatic injuries. In addition, 11.11% of patients had been victims of electrical trauma, highlighting the varied mechanisms underlying their wounds. Athanaselis et al. [17] reported that the site was the distal quarter leg at 43.3% and the heel at 10%.

De Blacam et al. [18] revealed steadily increasing usage of the sural flap for lower limb reconstruction, which may indicate a shift away from more complex and costly microsurgical techniques. This trend may be attributed to a growing recognition of the importance of cost-effective and resource-efficient wound management approaches, which may result in lower hospital stays.

Abdellah et al. [19] stated that wounds distribution across the lower limb was heterogeneous, with 11.1% of patients having defects in the middle third of the leg, 16.7% having defects over the distal third of the leg, and 5.6% having wounds affecting the dorsum of the ankle and foot and 5.6% having wounds affecting heel.

Daar et al. [20] performed a systematic review containing forty-three studies, 479 cases, and 481 flaps. They noted that the most common wound distribution was at the heel, 40.8%.

When raising the flap, the sural and peroneal communicating nerves are cut. At the same time, numbness in the area supplied by the sural nerve is not typically a significant concern for many surgeons, as it tends to resolve independently.

Aydin et al. [21] described that nerve-sparing technique for repairing foot and ankle defects, with promising results. However, the study had a small sample size and a relatively high rate of partial flap necrosis (20%). The technique was most reliable when the flap was designed in the lower two-thirds of the posterior calf. Al-Qattan [22] reported that patients who received a radial sensory flap without the sural pedicle experienced more complications compared to those who had the sural pedicle included.

Complications observed in this study were ankle stiffness in 3.85%, of patients were associated with distal tibial fractures, we believe that it could be more attributable to the fracture themselves rather than the flap procedure, distal necrosis in 1.92%, partial flap loss in 1.92%, superficial infection in 3.85%, and delayed healing in 7.69%.

The complication profile of the RSF demonstrated favorable outcomes, with a remarkable 82% of flaps showing no evidence of flap-related complications. The high rate of complication-free healing suggested that the RSF is a reliable and effective approach for soft tissue reconstruction [23]. The learning curve does not appear to influence complication rates, and a very weak correlation was found between surgeon experience and the failed reconstruction frequency, suggesting that other factors may play a more significant position in determining outcomes [24].

The success of the sural flap is closely linked to the meticulousness of the operative technique and the incorporation of refinements to the original description, which have been implemented to improve venous outflow and enhance flap viability [25].

Singh et al. [15] noted that complications encountered after RSF were partial flap marginal necrosis in 18.75%, venous congestion in 25%, donor site partial necrosis in 12.5%, and donor site complete necrosis in 6.25%.

Clivatti et al. [16] revealed that 44.4% of patients experienced complications, with partial necrosis being the most common complication (75%) and distal epitheliosis being less common (25%).

Athanaselis et al. [17] noticed that among the successfully healed flaps, 24% developed partial necrosis, and 20% failed to recover.

Abdellah et al. [19] demonstrated that infections affect 16.7% of patients, distal ischemia in 8.35%, and total flap loss in 2.8%. Notably, using the modified sural flap was associated with improved flap viability, which may have contributed to reducing the incidence of complications.

Daar et al. [20] revealed that the partial and total flap loss rates were 15.4% and 3.1%, respectively, while the overall complication rate was 33.7%.

One of the primary constraints of this investigation was the limited scope of the participant pool, which was comprised of a relatively small number of cases. Furthermore, the study was restricted to a single institutional setting, which may have introduced biases and limited the generalizability of the findings.

## Conclusion

In conclusion, reversed sural flaps offer a compelling alternative to free flaps, particularly for elderly patients with comorbidities. Their inherent blood supply chain makes them a safer and potentially less complex option, reducing the risk of complications at both the donor and recipient sites. Additionally, a fasciocutaneous tail at the pedicle mitigates the risk of venous obstruction and kinking. Reversed sural flaps demonstrate high resilience, making them well-suited for soft tissue defect reconstruction in the distal leg, ankle, heel, and foot areas that can be particularly challenging. Furthermore, reversed sural flaps can provide a valuable solution for post-surgical wound dehiscence, offering salvageable tissue for successful closure.

## Data Availability

Data is available upon reasonable request from corresponding author.
